# Differential SINE evolution in vesper and non-vesper bats

**DOI:** 10.1186/s13100-015-0038-4

**Published:** 2015-05-15

**Authors:** David A Ray, Heidi JT Pagan, Roy N Platt, Ashley R Kroll, Sarah Schaack, Richard D Stevens

**Affiliations:** Department of Biological Sciences, Texas Tech University, Lubbock, TX 79409 USA; Harbor Branch Oceanographic Institute, Florida Atlantic University, Fort Pierce, FL USA; Department of Biology, Reed College, Portland, OR 97202 USA; Department of Natural Resources Management and the Museum, Texas Tech University, Lubbock, TX 79409 USA

## Abstract

**Background:**

Short interspersed elements (SINEs) have a powerful influence on genome evolution and can be useful markers for phylogenetic inference and population genetic analyses. In this study, we examined survey sequence and whole genome data to determine the evolutionary dynamics of Ves SINEs in the genomes of 11 bats, nine from Vespertilionidae.

**Results:**

We identified 41 subfamilies of Ves and linked several to specific lineages. We also revealed substantial differences among lineages including the observation that Ves accumulation and Ves subfamily diversity is significantly higher in vesper as opposed to non-vesper bats. This is especially interesting when one considers the increased transposable element diversity of vesper bats in general.

**Conclusions:**

Our data suggest that survey sequencing and genome mining are valuable tools to investigate SINE evolution among related lineages and can provide substantial information about the ability of SINEs to proliferate in diverse genomes. This method would also be a useful first step in determining which subfamilies would be the best to target when developing SINEs as markers for phylogenetic and population genetic analyses.

**Electronic supplementary material:**

The online version of this article (doi:10.1186/s13100-015-0038-4) contains supplementary material, which is available to authorized users.

## Background

Now that it is known that transposable elements (TEs) comprise a significant proportion of most multicellular eukaryotic genomes, there is great interest in understanding their patterns of proliferation and the factors determining their relative success across various lineages. Two classes of TEs are delineated according to mobilization mechanism. Class I elements, the retrotransposons, move through an RNA intermediate, allowing the original copy to stay in place, resulting in replicative gains in copy number. Most Class II elements, the DNA transposons, mobilize in DNA form, with one subclass (hAT, piggyBac, and so on) relying on excision and re-integration (cut-and-paste) and a second subclass (Helitrons and Mavericks) utilizing a DNA-based replication mechanism. In mammals, retrotransposons are by far the most active and largest class of repetitive sequences. This is exemplified by the high prevalence of long and short interspersed elements (LINEs and SINEs) in the human genome, where the primate SINE, *Alu*, has reached over one million copies and continues to multiply [1]. For the last 40 million years, TE activity in mammals has been limited almost exclusively to Class I elements [2-7]. However, exceptions have been identified in several mammals, where multiple horizontal transfers of Class II elements have occurred, and/or activity levels of DNA transposons are high [8-14].

SINEs have been shown to influence genomes in multiple ways including the introduction of CpG islands, regulatory motifs, and as the substrate for homologous and non-homologous recombination events (reviewed in [15]). In addition to their impacts on genome structure and function, they have also proven to be exceptionally useful genetic markers, particularly in the elucidation of phylogenies [16-21]. Once inserted, SINEs are rarely excised [22] and, after fixation in the population, will be vertically inherited, becoming shared derived characters. Further, the absence of a SINE insertion at any particular locus can be safely assumed to represent the ancestral condition. However, because SINE subfamilies will emerge, multiply, and eventually die out over a finite period, it is critical to identify the subfamilies that were active during the period of interest for the phylogeny being inferred. In that way, the researcher is more likely to identify insertions that will be informative in such analyses.

The confident identification of phylogenetically informative patterns requires large numbers of SINE insertions, preferably from multiple representatives of the clade of interest. The most efficient way to identify such patterns would be to query representative genome drafts. While genomes are being assembled at an increasing rate, this is not feasible for all groups. Instead, one can often hope for at best a single genome sequence from the clade of interest, and for most clades even that is not available. An alternative strategy would be to take advantage of high-throughput sequencing technologies and survey sequencing a group of related genomes. Such survey sequencing would provide large amounts of potentially informative data on the identity of TE subfamilies in a range of genomes for a relatively low cost [23-26].

Ves is a tRNA-derived SINE family found in yangochiropteran bats, a clade that includes all microbats with the exception of the yinpterochiropteran microbats of families Megadermatidae, Rhinolophidae, and Rhinopomatidae [27,28]. For this study, we examined Ves accumulations in the draft genomes of the vesper bats *Eptesicus fuscus*, *Myotis brandti*, *M. davidii*, *M. lucifugus*, and the non-vesper bat *Pteronotus parnellii*. We also examined survey data collected from the genomes of six other bats, the verspertilionids, *Corynorhinus rafinesquii*, *Lasiurus borealis*, *M. austroriparius*, *Nycticeius humeralis*, *Perimyotis subflavus*, and the phyllostomid *Artibeus literatus*. Our results demonstrate differential activity among these lineages and allow us to identify the subfamilies that are most likely to be informative at various branches within the yangochiropteran phylogeny. We also developed a method for determining lineage specificity of SINE subfamilies and, using this method, were able to establish subfamily identities within each taxon and identified several instances of lineage-specific Ves activity.

## Results

To identify patterns of Ves activity in the sampled bats, we re-analyzed the survey-sequence data of Pagan *et al*. [25] and performed a *de novo* analysis of SINEs in the draft genomes of *Eptesicus fuscus*, *Myotis brandtii*, *M. davidii*, *M. lucifugus*, and *Pteronotus parnellii* (AAPE00000000, ANKR00000000, ALWT00000000, ALEH00000000, and AWGZ00000000, respectively). The survey data consisted of approximately 1.3 million 454 reads from six bats. These reads averaged approximately 300 nt in length and represented between 0.76% and 4.75% of the sampled genomes. Complete details are available in Table 1 and Pagan *et al*. [25]. Ves family SINEs were identified in all of the taxa examined. The two non-vesper bats, *A. lituratus* (Phyllostomidae) and *P. parnellii* (Mormoopidae), exhibited lower overall Ves content, suggesting lower levels of accumulation in those lineages. Conversely, *N. humeralis* exhibited higher than average Ves accumulation than its fellow vespertilionids. This is likely due to the presence of two novel subfamilies that are not found in other vesper bats (see below). This pattern suggests that the subfamilies evolved after the split between the *N. humeralis* ancestor and the remainder of Vespertilionidae and contributed significantly to genome content in the lineage leading to *Nycticeius*.

**Table 1:** **Taxa examined in this study, data used and basic statistics describing Ves content and Ves insertions used for our analysis of subfamilies**

**Taxon**	**Abbreviation**	**Family, subfamily**	**Data source**	**Bases queried**	**Total Ves bases identified**	**% Ves-derived bases**	**Total Ves fragments identified**	**Full-length Ves insertions analyzed in COSEG**
*Artibeus lituratus*	Alit	Phyllostomidae, Stenodermatinae	SS	101,137,176	2,510,325	2.48%	18,038	5,964
*Corynorhinus rafinesquii*	Craf	Vespertilionidae, Vespertilioninae	SS	108,013,590	4,191,322	3.88%	33,888	8,068
*Eptesicus fuscus*	Efus	Vespertilionidae, Vespertilioninae	WGS	388,423,918	17,126,373	4.41%	97,440	15,000
*Lasiurus borealis*	Lbor	Vespertilionidae, Vespertilioninae	SS	71,255,672	3,431,237	4.82%	25,494	7,624
*Myotis austroriparius*	Maus	Vespertilionidae, Myotinae	SS	21,992,128	1,078,155	4.90%	8,720	2,143
*M. brandtii*	Mbra	Vespertilionidae, Myotinae	WGS	533,920,351	28,106,987	5.26%	160,836	15,000
*M. davidii*	Mdav	Vespertilionidae, Myotinae	WGS	420,014,318	23,723,693	5.65%	137,294	15,000
*M. lucifugus*	Mluc	Vespertilionidae, Myotinae	WGS	497,001,341	23,480,719	4.72%	129,921	15,000
*Nycticeius humeralis*	Nhum	Vespertilionidae, Vespertilioninae	SS	34,287,171	2,250,938	6.56%	18,329	4,218
*Perimyotis subflavus*	Psub	Vespertilionidae, Vespertilioninae	SS	29,578,028	1,290,520	4.36%	10,669	2,419
*Pteronotus parnellii*	Ppar	Mormoopidae	WGS	265,642,944	5,805,924	2.19%	35,450	15,000

SS = survey sequence data, WGS = draft genome data.

Ves elements spanning at least 90% of their respective consensus sequences from each survey data set ranged from 2,143 in *M. austroriparius* to over 8,000 in *C. rafinesquii*. Including extracted Ves elements from genome drafts provided a total of 105,436 insertions to be analyzed. Our iterative approach to defining subfamilies (see ‘Methods’) resulted in a final Ves library consisting of 41 subfamily consensus sequences.

**Figure 1:**
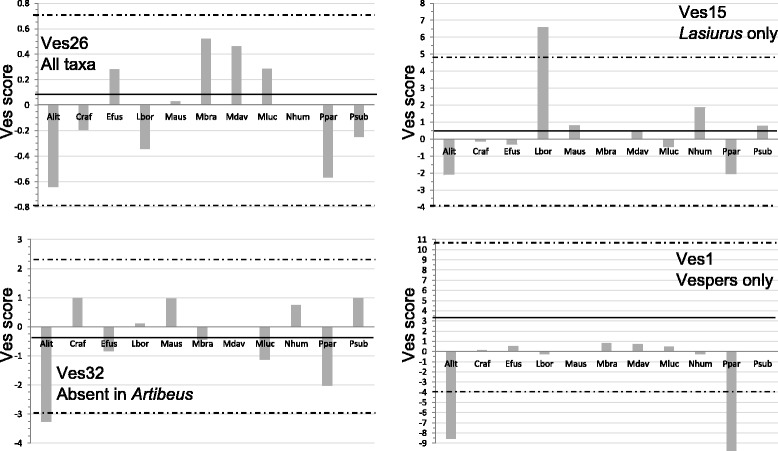
Representative Ves score plots used to identify lineage specific subfamilies. *Solid black horizontal lines* indicate the mean Ves score and *dashed black horizontal lines* indicate the two standard deviation upper and lower bounds. Taxon abbreviations are as in Table 1.

To determine potential lineage specificity, we developed a novel measure we refer to as the ‘Ves score’ for each putative subfamily. This score is a statistic consisting of the proportion nucleotides from each subfamily in each taxon compared to the median genome proportion (see ‘Methods’ for details). When scores for any individual taxon fell outside a range encompassing two standard deviations of the mean score (approximating *α* = approximately 0.05 on a normal distribution), the subfamily was considered specific to a given lineage. Ves score plots indicated several levels of lineage specificity and four examples are provided in Figure 1 and Additional file 1. Scores for Ves26 in all taxa fell within two standard deviations of the mean Ves score, indicating that this subfamily is present at approximately equal numbers in all taxa. By contrast, the score for Ves32 in *A. lituratus* fell below the two standard deviation threshold but within the two standard deviation range in all other bats, showing that this subfamily is present in all taxa except our representative phyllostomid. The score for Ves15 is higher than the cutoff in *L. borealis* but not in any other sampled bat, indicating that the subfamily is present in significantly higher numbers in this taxon. In addition to the single *L. borealis*-specific subfamily, Ves15, two *N. humeralis*-specific subfamilies were identified, Ves14 and Ves18 (Additional file 1). In the final example, Ves1, the subfamily is present in all vesper bats but absent in the two representative non-vespers. Not surprisingly given the taxon sampling, the majority of the subfamilies described are specific to vesper bats.

Visual examination of log plots suggested that several subfamilies might border on lineage specificity but not reach the 2.0 sigma cutoff. We relaxed our two standard deviation range requirements by increments down to sigma =1.5 (Additional file 1) and found that several subfamilies could be labeled as borderline lineage specific. For example, Ves23 and Ves31 could be considered as vesper bat specific if one lowers the threshold slightly to 1.9. This suggests that the method can be used as a first approximation to determine likely trends in the data regarding lineage specificity but that individual cases may require special attention.

By examining pairwise divergences among copies of each subfamily and assuming similar neutral mutation rates in bat lineages, it is possible to provide relative estimates of the accumulation periods for various groups of TEs in each lineage. Such estimates assume that element accumulation initially resulted in the formation of multiple identical copies of each retrotransposed element. As time passes, the initially identical elements diverge at a rate determined by the neutral mutation rate. Thus, within a given subfamily, higher average pairwise divergence values among its members indicate more time that has elapsed. So it follows that a subfamily with a higher average pairwise divergence was active in the more distant past than a family with lower average pairwise divergence.

**Figure 2:**
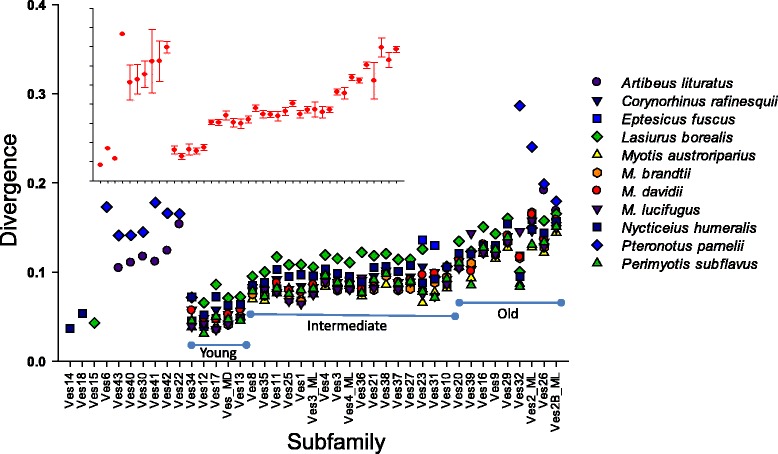
Average divergence values within taxa and among all taxa (inset) for each subfamily. The three major age categories identified among the most widely distributed subfamilies are indicated. *x*-axis values for the inset are the same as for the main figure.

Figure 2 shows the average divergences among subfamilies within and among all taxa. Within the most widely distributed subfamilies, there appear to be three general age categories. For subfamilies that are more restricted, two patterns emerged. First, the three subfamilies that are specific to *L. borealis* and *N. humeralis* (Ves14, 15, and 18) are young compared to most other subfamilies. This is expected given the relatively recent divergence of these genera from other vesper bats [29]. The converse is true for the subfamilies that are present in non-vesper bats, which diverged from Vespertilionidae approximately 45 mya and from each other approximately 34 mya [29]. These subfamilies show evidence of little recent accumulation, suggesting low rates of novel subfamily evolution and diversity. Indeed, if we consider only *M. lucifugus* and *P. parnellii*, two taxa for which we have full genome drafts, we observe that 28 subfamilies have evolved in the former lineage compared to only seven in the latter. Furthermore, if we consider average sequence divergence as a proxy for subfamily age, the subfamilies that are specific to the *P. parnellii* lineage evolved early and experienced little subsequent diversification, whereas novel subfamilies have been continuously evolving throughout the entire history of vesper bats (Figure 2).

**Figure 3:**
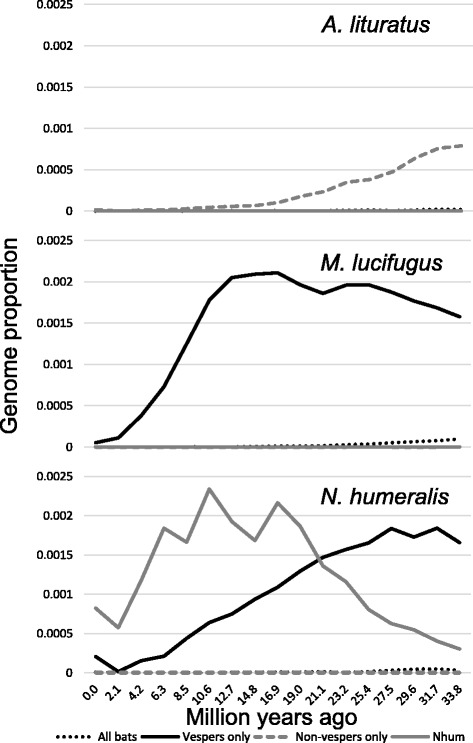
Temporal accumulation plots illustrating Ves contributions to three bat genomes over the past, approximately 34 my. In each case, genome proportions occupied by each category of the Ves subfamily are plotted against periods of accumulation.

We were also interested in whether or not Ves has been accumulating at similar rates in vesper and non-vesper bats. To examine if the proportion of Ves-derived bases were significantly different between vespertilionid and noctilionoid (*A. lituratus* and *P. parnellii*) groups, we conducted an independent sample *t*-test based on arcsine transformed proportions. The vespertilionid mean percent Ves-derived bases (Table 1) was larger than the noctilionoid mean, and this difference was significant (*t* = 5.33, df = 9, *P* < 0.001). Thus, vesper bats have experienced significantly higher rates of Ves accumulation compared to the two non-vesper bats in the study. The temporal accumulation plots in Figure 3 and Additional file 2 illustrate substantial differences in Ves accumulation within and among lineages. Within Vespertilionidae, there is less variability in accumulation patterns, with the exception of *N. humeralis*, whose genome has accumulated substantial mass from the two lineage-specific subfamilies we detected.

**Figure 4:**
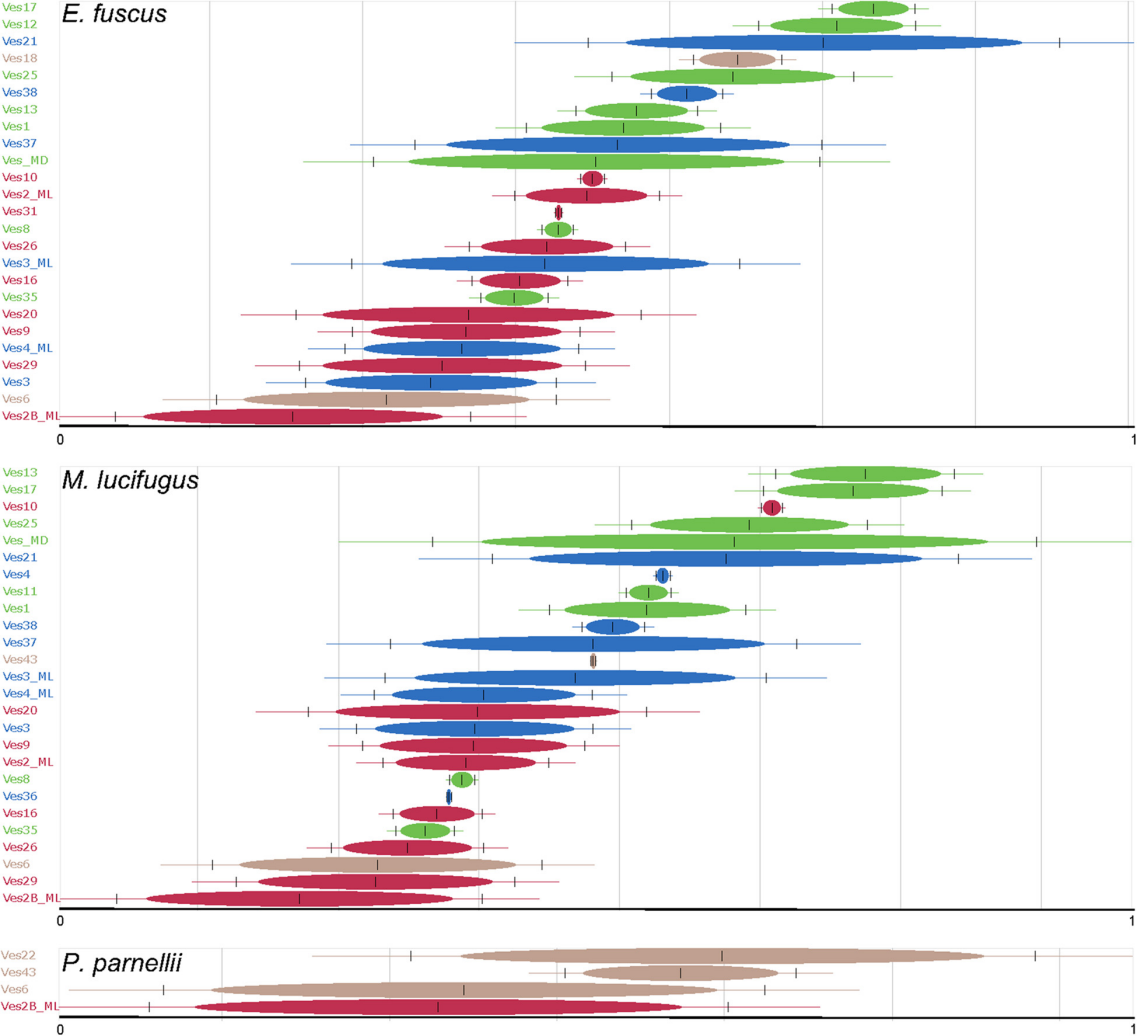
TinT results for three taxa. *Green*, *blue*, and *red bars* represent subfamilies identified in Figure 4 as young, intermediate, and old, respectively. *Brown bars* indicate subfamilies that are less widely distributed. Plots for *M. brandtii* and *M, davidii* are available in Additional file 3. Numbers on the *x*-axis are relative time periods within each taxon with zero at the origin of detectable activity for these SINE subfamilies and one representing the boundary of current activity.

Transposition in transposition (TinT) analysis uses nested insertion analysis to independently estimate relative periods of activity among TE families [30]. Because of the limitations of survey sequence data, including the relatively small average read length, TinT analysis was limited to the genome drafts. Analyses of these genomes using a custom Ves subfamily library confirm the patterns suggested by investigations of lineage specificity and genetic divergence with subfamilies categorized as ‘young,’ ‘intermediate’, or ‘old’ generally falling at the top, middle, or bottom of the TinT plots, respectively (Figure 4, Additional file 3). Interestingly, Ves18, which was identified as being specific to the *N. humeralis* lineage appears in the *M. brandtii* and *E. fuscus* TinT plots. This can be explained by assuming that the Ves18 lineage began accumulating at low rates in a common ancestor of the broader clade but did not achieve substantial retrotranspositional success until after the *Nycticeius* lineage diverged from the remainder of Vespertilioninae. Indeed, our analysis of lineage-specificity suggests that Ves18 is present at low levels in all of the other bats we investigated, with genome proportions ranging from 0.001% to 0.069%, compared to 0.424% in the *N. humeralis* genome (Additional file 1).

**Figure 5:**
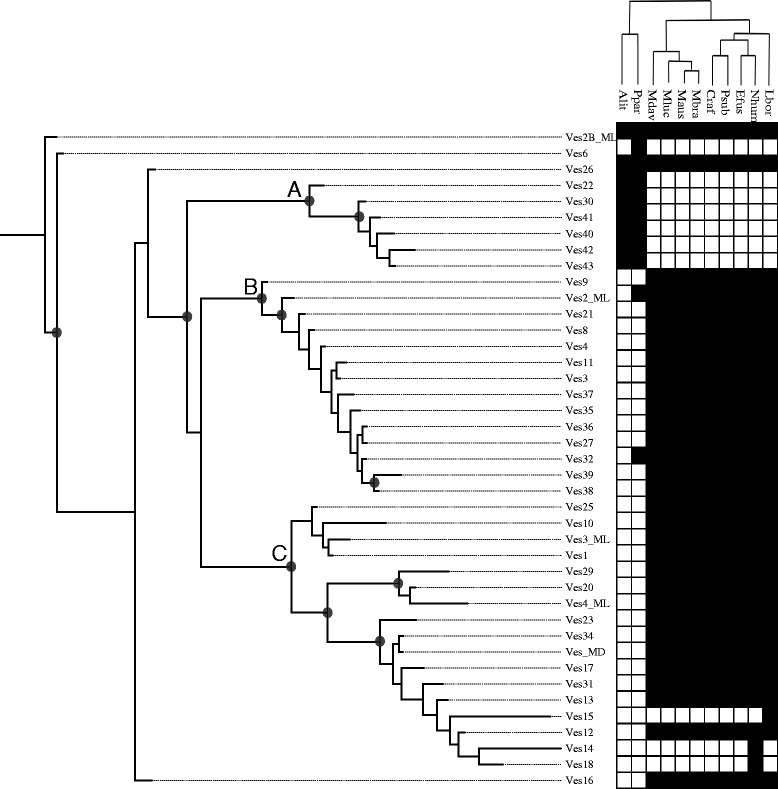
Bayesian tree of Ves subfamily relationships. *Gray circles* represent nodes with posterior probabilities of 0.95 or greater. The chart to the right indicates the species in which each subfamily can be identified as per the lineage specificity plots in Additional file 1. The cladogram at the top illustrates relationships among taxa as per Lack and Van Den Bussche (2010). Taxon abbreviations are as in Table 1.

Figure 5 illustrates the relationships among subfamilies recovered by Bayesian analysis of the core consensus sequences of each subfamily. Because subfamilies 26 and 2B_ML are present in all of the bats analyzed, they likely represent basal Ves lineages, and Ves2B_ML was judged to be the older of the two by TinT and our genetic distance estimates (Figures 2 and 4). We therefore rooted the tree on that subfamily. The resulting topology suggests three well supported Ves lineages. Clade A consists of six subfamilies that are restricted to non-vesper bats. Clade B consists, with two exceptions, of subfamilies that are specific to vesper bats. The two exceptions (Ves2_ML and Ves32) are found in *P. parnellii* but do not show significant accumulations in *A. lituratus* (Additional file 1). This pattern is unexpected given the fact that these taxa belong to the sister families Moomopidae and Phyllostomidae. In both cases, however, these subfamilies exhibit substantially, but not significantly, lower rates of accumulation in the non-vesper data (see Ves32 in Figure 1). This suggests that the two subfamilies were experiencing reduced retrotransposition in both lineages, consistent with the observation of reduced accumulation described above but that these two subfamilies may have managed to replicate a bit more successfully in the lineage leading to *Pteronotus* after its divergence from the ancestor of *Artibeus*. Clade C is similar in that it also comprises vesper-specific subfamilies, but it also harbors the three subfamilies that are specific to the *Nycticeius* and *Lasiurus* lineages with the two *Nycticeius* subfamilies being sister to one another. The picture to be gleaned therefore is that our subfamilies generally reflect the phylogeny of the bats whose genomes they inhabit.

## Discussion

Two of our observations, increased Ves diversity and increased Ves accumulation in Vespertilionidae, are interesting in the context of overall mammalian and chiropteran TE diversity. As has been repeatedly observed [25,31,11,12], vesper bats are home to an astonishing diversity of DNA transposons not seen in any other mammal to date. Furthermore, these DNA transposons appear to have led to functional evolutionary innovations [14,13]. Increased Ves and DNA transposon accumulation appears to be a characteristic of this family, which is the second most species-rich mammalian clade, and may have played a role in its diversity.

The data presented here suggest that several subfamilies would serve as excellent markers for investigating relationships within bats. For example, for those interested in early divergences among all yangochiropterans, probing for members of Ves2B_ML or Ves26 would be most appropriate. For researchers interested in investigations of relationships within the lineage leading to genus *Nycticeius*, it would be preferable to focus on Ves14 and/or Ves18. Broader interest in the evolution of Vespertilionidae/Vespertilioninae would be served by focusing on any of the intermediate subfamilies.

It should be pointed out that survey sequencing was accomplished using 454/Roche chemistry. When the analysis was first conceived, this chemistry was the only one available that would provide reads long enough to sequence full-length Ves insertions. However, Illumina chemistry has recently achieved read lengths of 300 nt on some of its systems. Using a paired-end sequencing strategy and creating libraries consisting of fragments under 600 nt would produce overlapping reads of more than sufficient length to accomplish the same task but resulting in much larger data sets than the one described here. For example, we recently surveyed several mammal genomes as part of a project to investigate LINE activity using this strategy and obtained just under one million paired-end reads using 1/10th of a MiSeq lane (Mangum *et al*., unpublished data). In our original study, we required multiple full 454 runs to obtain the just under 1.3 million reads of similar length [25]. This suggests that substantially larger data sets, potentially consisting of much larger numbers of taxa, could be easily and inexpensively obtained.

Indeed, we recently used the information provided by this study to inform a novel experimental protocol to analyze the phylogeny of selected *Myotis* bats (Platt *et al*., under revision). In that study, a combined, computational and laboratory-based approach based on ME-Scan [32] was used to identify potentially polymorphic SINE insertions in seven species. Probes were designed that match subfamilies in Clade C. That work was successful in inferring previously established relationships among the bats investigated, further suggesting that this method will be useful in informing projects designed to use SINEs as phylogenetic markers.

## Conclusions

While genome sequencing costs continue to decline, the huge biological diversity observed still prevents us from achieving the ideal - a complete genome from all taxa. We find that this survey method can provide substantial information about SINE families/subfamilies in a range of taxa at minimal cost and suggest that it may serve as a valuable initial step in guiding SINE-based analyses of a variety of taxa, especially those that are not represented by a draft genome. Furthermore, the Ves score method we developed to identify lineage-specific subfamilies should be easily implemented in studies of SINE families in a wide variety of taxa.

Finally, there is no reason to limit the methods described here to SINEs alone, and substantial information about the overall TE content in a genome can also be gleaned.

## Methods

We used RepeatMasker [33] to query all survey sequence data and approximately one quarter of the whole genome drafts. We used a custom Ves library consisting of the VES, Ves2_ML, Ves2B_ML, Ves3_ML, and Ves4_ML subfamilies from RepBase [34]. All Ves insertions spanning at least 90% of the identified consensus were extracted, limiting ourselves to 15,000 hits from the genome drafts. The extracted sequences were combined into a single set of Ves insertions and analyzed using COSEG [35,36] after aligning them to the VES4_ML consensus sequence. A custom Perl script provided by R. Hubley was used to refine the consensus sequence for each Ves subfamily and is available upon request.

Upon identification of Ves subfamily structure using COSEG, a custom RepeatMasker library was constructed and applied to a pseudogenome consisting of all survey sequences and the original subset of WGS data. To verify the presence of each subfamily in the data, 25 random hits identified as belonging to each subfamily were extracted and aligned with their respective consensus. Alignments were examined by eye and, when necessary, new 50% majority-rule consensus sequences were generated. These new consensus sequences were compared among themselves and with the original RepBase Ves elements. Several predicted subfamilies were collapsed into identical subfamilies already defined in RepBase or into other COSEG-derived subfamilies after generating refined consensus sequences. Analysis of two subfamilies, 7 and 33, revealed that these are instances where a Ves element inserted into an active Helitron element, which then deposited copies throughout the genome as it multiplied. Because these two predicted subfamilies were likely disseminated throughout the genome by mechanisms other than retrotransposition, they were not included in subsequent analyses of SINE dynamics. For any COSEG-predicted subfamilies matching those already described in RepBase, the RepBase subfamily designations were used. All newly described subfamily consensus sequences are available in Additional file 4 and have been deposited in RepBase.

The 3’ ends of Ves elements consist of an A-rich region preceded by multiple low complexity, pyrimidine-rich regions. These regions are highly variable and, thus, problematic for estimating divergence in downstream analyses. Thus, we created a second library consisting of Ves ‘core’ sequences (defined as the 5’ ends up to but not including the first major poly-pyrimidine tract). These core sequences averaged 159 bp in length compared to the average 212 bp for the full-length library. Relationships among Ves subfamilies were inferred by generating a Bayesian tree of the core consensus sequences in MrBayes v3.2.1 [37]. We used the GTR model of nucleotide substitution and performed one million iterations with a burnin of 1000.

To determine potential lineage specificity of Ves subfamilies, we first determined the genome proportions occupied by each subfamily using RepeatMasker. The total number of bases assigned to each Ves subfamily in each data set was then divided by the total number of bases analyzed from each taxon. For each subfamily, the median genome proportion among the eight taxa was calculated. We next calculated the Ves score, log_2_ (proportion/median), for each subfamily within each taxon and compared these by calculating the mean Ves score for each subfamily among the taxa. Ves scores within each taxon were plotted and compared to the mean scores for each subfamily. When scores for any individual taxon fell outside a range encompassing two standard deviations of the mean score (approximating *α* = approximately 0.05 on a normal distribution), the subfamily was considered a candidate for lineage specificity, with the home lineage(s) being determined based on its presence or absence in the species under consideration.

To calculate approximate periods of accumulation we used a modified version of the calcDivergenceFromAlign.pl script that is included in the RepeatMasker package to calculate Kimura two-parameter distances between each insertion and its respective consensus [10]. The -noCpG option was invoked. We applied the mutation rate estimated by Ray *et al*. [11], 2.366 × 10^−9^ substitutions per site/my to calculate average divergences among subfamily insertions and within taxa and to plot relative accumulation periods.

Temporal analyses were supplemented by implementing TinT (Transposition in transposition) analyses using the online server at http://www.bioinformatics.uni-muenster.de/tools/tint/ [30,38]. For this analysis, we queried the full genome drafts of three *Myotis* species, *E. fuscus*, and *P. parnellii* using our custom Ves library and generated bar graphs to illustrate rates of Ves elements inserting into other Ves elements, a proxy for relative activity periods.

## Additional files

Additional file 1:
**Ves score calculations and plots.**
Additional file 2:
**Ves accumulation plots.**
Additional file 3:
**TinT plots for all taxa.**
Additional file 4:
**Consensus sequences of all Ves SINEs.**

## Abbreviations

TE: transposable element
LINEs: tong interspersed elements
SINEs: short interspersed elements
TinT: transposition in transposition
