# Supplementary figures and images for: Differential SINE evolution in vesper and non-vesper bats
Source: Mob DNA. 2015 May 15;6:10. doi: 10.1186/s13100-015-0038-4 (PMC4436864; doi:10.1186/s13100-015-0038-4)

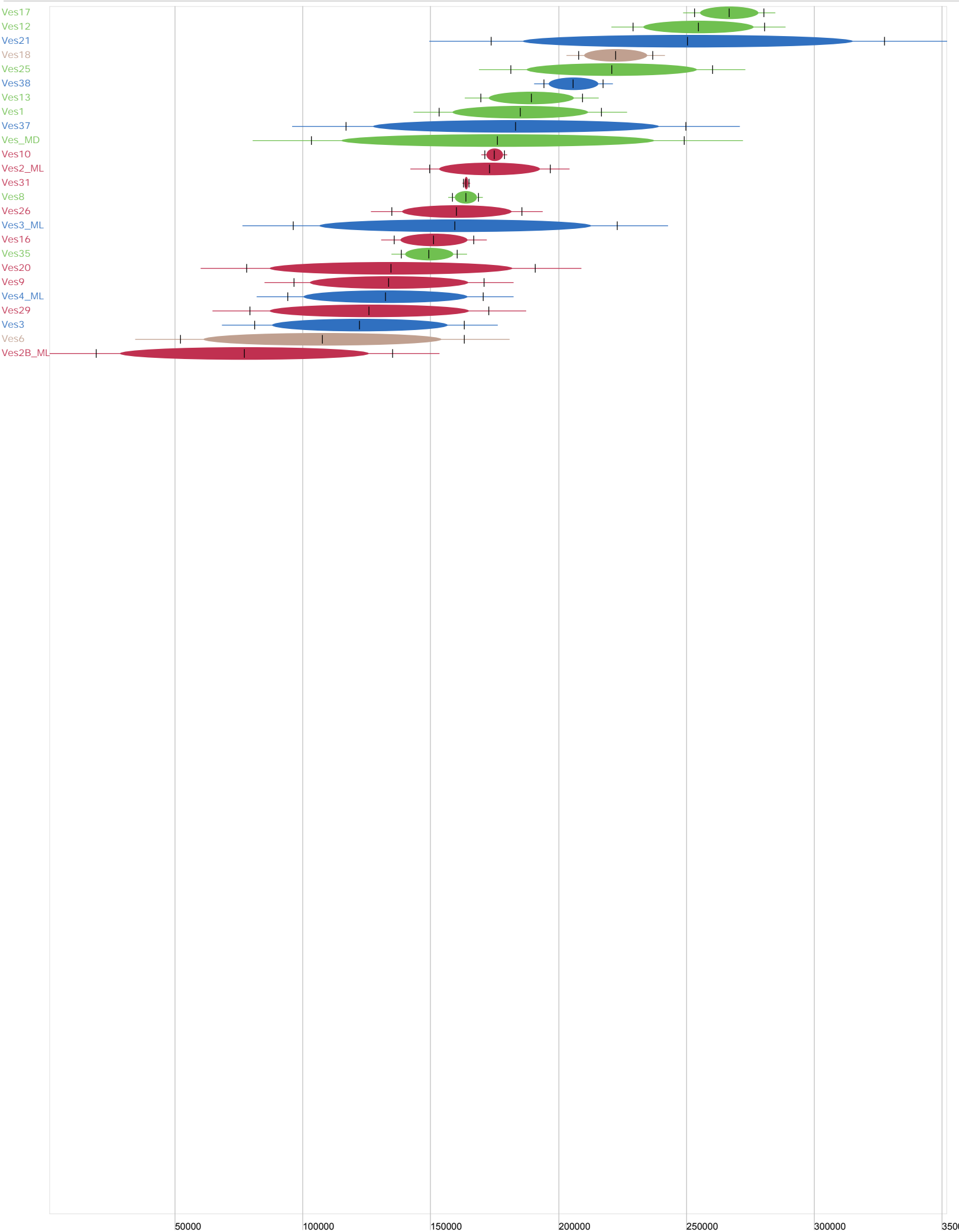

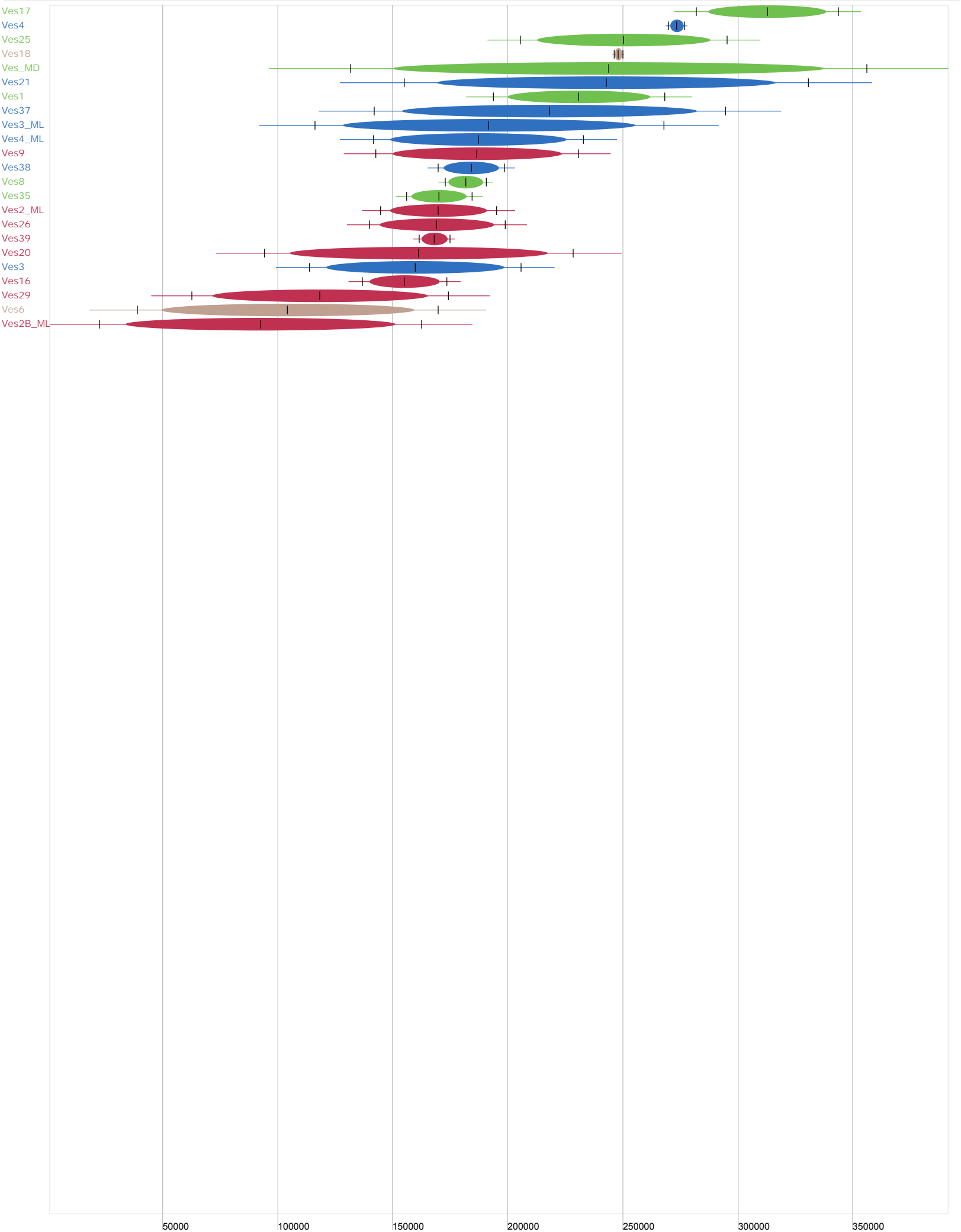

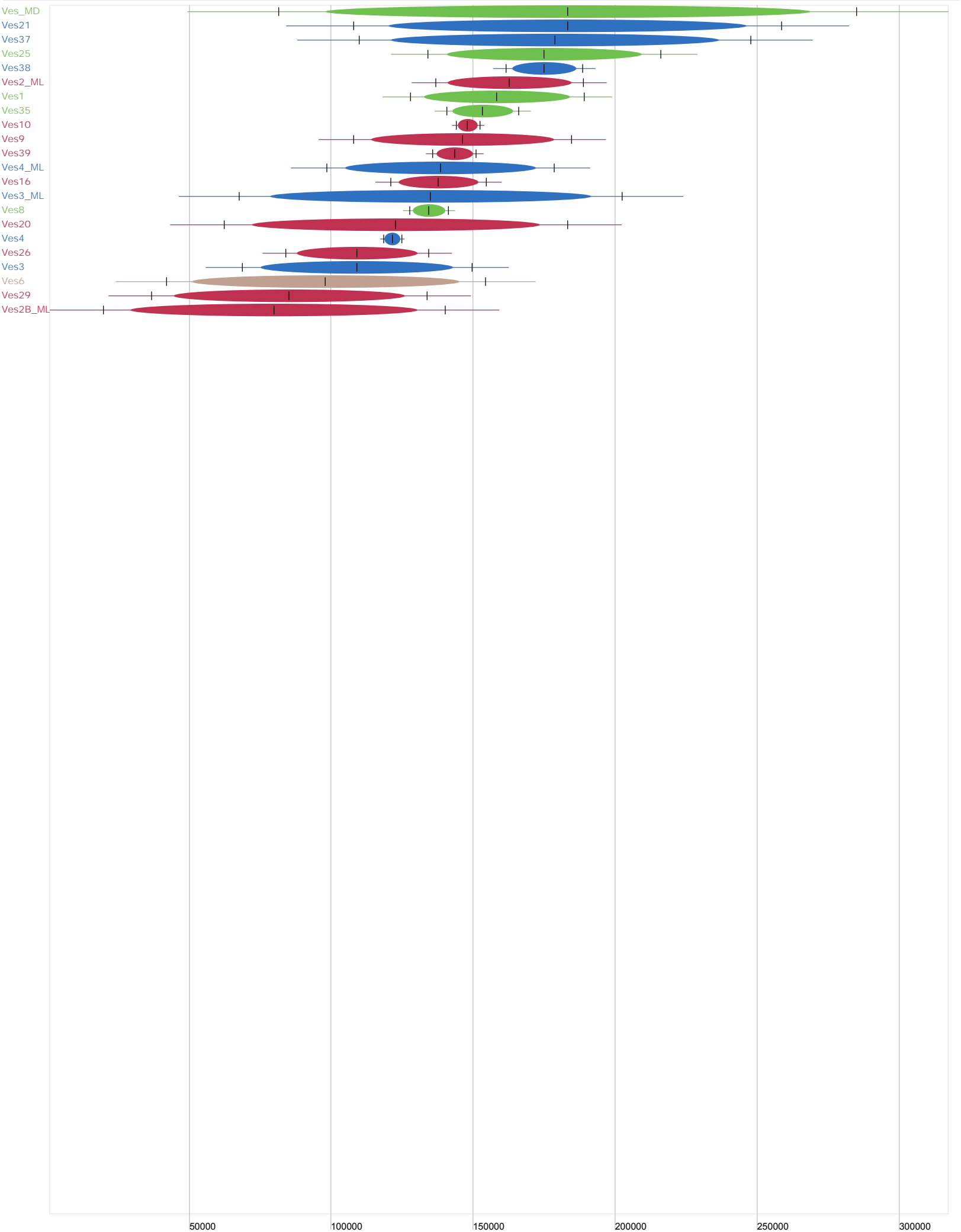

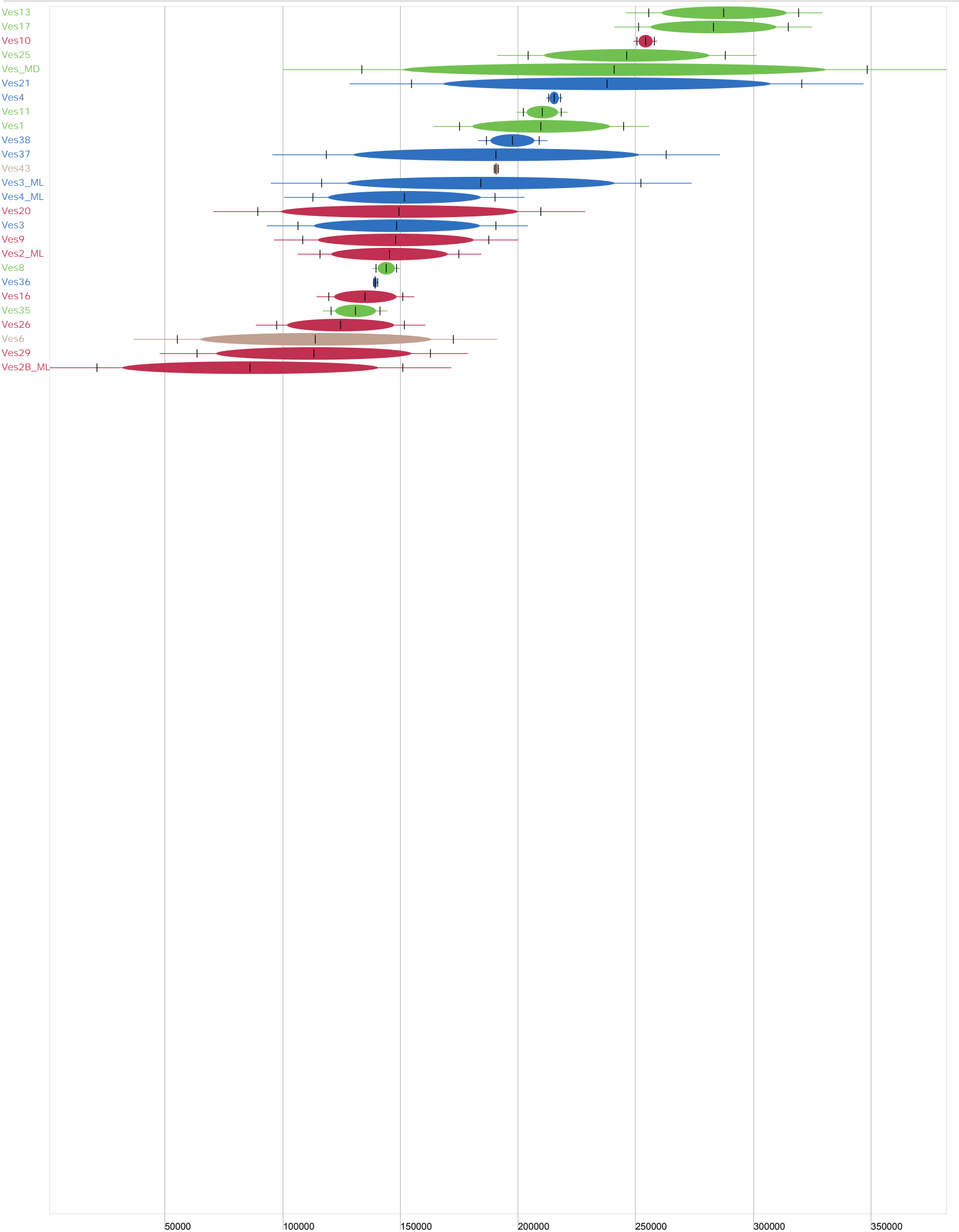

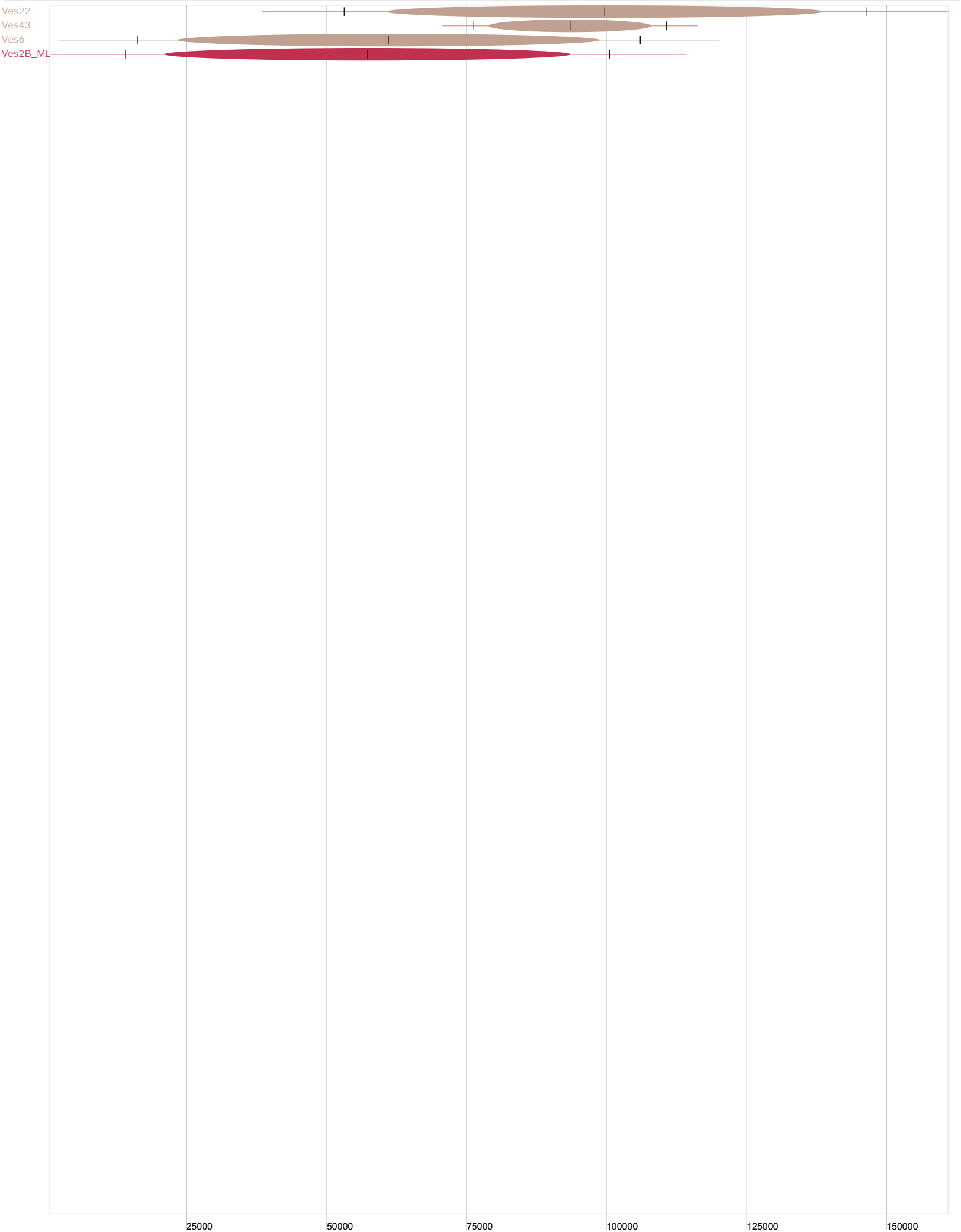

Supplement: Additional file 3: — TinT plots for all taxa. [file 13100_2015_38_MOESM3_ESM.pdf]
